# Show Me Competence or Make Me Feel Warm: The Impact of Green Brand Anthropomorphism on Consumers’ Purchasing Intentions

**DOI:** 10.3390/bs16030316

**Published:** 2026-02-25

**Authors:** Sinan Li, Haoyuan Chang, Jin Ma, Kai Chen

**Affiliations:** 1 School of Economics and Management, Beijing Forestry University, Beijing 100083, China; lisinan@bjfu.edu.cn; 2 People’s Education Press, Beijing 100081, China; changhy@pep.com.cn; 3 College of Education for the Future, Beijing Normal University at Zhuhai, Zhuhai 519085, China

**Keywords:** green brand anthropomorphism, purchasing intentions, psychological distance, product attributes, influence mechanism

## Abstract

Green consumption is a key path to dealing with environmental problems and promoting sustainable development. Green brand anthropomorphism can build an emotional bond between green brands and consumers by giving brands human characteristics, providing a new framework for promoting green consumption. This study uses a between-subject experimental design to explore the impact of green brand anthropomorphism on consumers’ purchasing intentions. Study 1 constructed an experimental framework of the impact of anthropomorphism (competence-oriented vs. warmth-oriented) on purchasing intentions and introduced non-anthropomorphism as a control group. It was found that brand anthropomorphism could improve consumers’ green purchasing intentions more than non-anthropomorphism, and the overall effect of warmth-oriented anthropomorphism was better than that of competence-oriented anthropomorphism. It further confirmed the mediating role of psychological distance, that is, green brand anthropomorphism shortened the psychological distance between consumers and brands, thereby improving purchasing intentions. Study 2 adopted a 2 (anthropomorphism: competence-oriented vs. warmth-oriented) × 2 (product attributes: functional vs. hedonic) experimental framework to reveal the moderating role of product attributes. It was found that functional attributes were suitable for competence-oriented anthropomorphism, and hedonic attributes were suitable for warmth-oriented anthropomorphism, which could more effectively improve green purchasing intentions. It was further verified that the mediating effect of psychological distance was regulated by product attributes. This study verifies the applicability of the Stereotype Content Model in the field of green consumption, addressing the gap in green brand anthropomorphism research regarding insufficient evidence on the differences between “anthropomorphism types (competence-oriented vs. warmth-oriented)” and their underlying mechanisms. It further reveals how psychological distance serves as a key behavioral mechanism linking brand stimuli to consumers’ green purchasing decision-making processes. Through two studies, it validates the effects of anthropomorphism types and their mediating mechanisms while also providing evidence for the moderating role of product attributes. This contributes a clearer explanatory framework for advancing brand anthropomorphism theory and informing sustainable marketing practices within the green consumption context.

## 1. Introduction

Climate change and carbon emissions are severe environmental problems facing the world. Green consumption has become an important demand-side force for reducing carbon emissions and promoting sustainable development (Guan et al., 2024). How to effectively promote consumers’ green purchases has also become an important direction for related research. Brands are an important factor influencing consumer behavior, and improving consumers’ willingness to purchase green products through green brand transformation has become a strategic choice for many companies (Lukin et al., 2022; Kautish et al., 2020). A green brand is usually defined as a brand that provides more significant ecological advantages than its competitors and increases consumers’ green purchase priority (Hartmann et al., 2005). Under the dual effect of policy advocacy and market promotion, consumers’ attention to green products has increased significantly, and green brands have gradually become the key to corporate competition (Watson et al., 2024; Chen et al., 2024). This is because green brands are a key component of brand identity and value creation, not only carrying functional values such as energy saving, environmental protection, health, and safety, but also symbolizing sustainable concepts (H. C. Wu et al., 2018; Y. C. Huang et al., 2014). While providing products to consumers, they are of enormous significance for alleviating resource consumption and reducing environmental pressure (Mehraj & Qureshi, 2022). As a market symbol that takes into account both environmental benefits and consumer satisfaction, the core value of a green brand lies in guiding consumers to make environmentally friendly choices and alleviating the burden of the ecological environment through sustainable practices (Baah et al., 2025), which helps to establish a more responsible and good brand image in the minds of consumers (Yadav & Pathak, 2016).

However, due to the limited popularity of the concept of green consumption, consumers’ understanding of green brands often stays at a shallow level (Janßen & Langen, 2017), the attributes of green labels are not clear (Daugbjerg et al., 2014), the credibility of green information is lacking (Verleye et al., 2023), and the emotional connection with green brands is weak (Wang et al., 2022). These challenges make consumers’ perception of the value of green brands insufficient. In addition, greenwashing will significantly reduce brand trust and negatively affect the reputation of green products (Sajid et al., 2024), resulting in a gap between green purchase intention and behavior (Witek & Kuźniar, 2023), which restricts the further expansion of the green consumption market and makes green purchase behavior difficult to form a stable market inertia (X. Liu, 2024). Against this backdrop, how to convey the value of green brands to consumers in a more intuitive and emotionally resonant way and promote the spread of green brands and changes in consumer behavior has become a focus of common concern for academia and industry.

As an effective marketing communication strategy, brand anthropomorphism can enhance the emotional appeal and cognitive accessibility of brands and is regarded as an effective strategy to improve brand communication effects and strengthen consumer connection (T. Kim et al., 2020; L. W. Yang et al., 2020). Brand anthropomorphism gives brands human-like characteristics, making them appear to be “thoughtful, emotional, and individualistic,” thereby making abstract brand concepts concrete and approachable, which helps break down the communication barriers between brands and consumers and provides a new path for the value transmission of green brands (H. Yang et al., 2023; Qin et al., 2025). Existing studies have shown that anthropomorphism can help brands stand out from homogeneous competition, make it easier for consumers to understand brand propositions, and form deeper emotional connections in interactions (Tahir et al., 2024). In particular, for green brands that emphasize safety, environmental protection, and sustainable concepts, anthropomorphism can play a role in different situations, effectively making up for the shortcomings of “strong professionalism and long distance” in green communication, providing more detailed strategic choices for green marketing, and allowing consumers to contact and understand green brand values in a more natural and acceptable way (Lu et al., 2021). However, existing studies have focused more on the binary comparison of “anthropomorphism or not” (May & Monga, 2014) and explored the impact of brand anthropomorphism on consumer attitudes, brand attachment, emotional response, and purchase intention. Although most studies have confirmed that anthropomorphism can improve green brand advocacy (Syahrivar et al., 2024), enhance green repurchasing intentions (Sehgal et al., 2023), and promote environmentally friendly behaviors (Zhou & Wang, 2022), some studies have also pointed out that brand anthropomorphism will become a negative marketing strategy in the consumption situation of pursuing uniqueness (Puzakova & Aggarwal, 2018). Therefore, the positive or negative role of brand anthropomorphism in promoting consumption needs further study. In addition, the academic community’s attention to anthropomorphized green brand types is still limited, and few studies have deeply analyzed the influence mechanism and boundary conditions of different types of anthropomorphism on green consumption behavior. Existing research still lacks systematic evidence regarding the mechanisms and boundary conditions of differences in anthropomorphism types (competence vs. warmth) in green consumption contexts, particularly lacking integrated testing within the psychological distance mechanism and product attributes matching framework. Since green consumption has both functional and moral attributes (B. Wu & Yang, 2018), consumers’ decisions are not only driven by product performance but also affected by factors such as emotion perception (Zhao et al., 2025), green trust (Borah et al., 2024), environmental knowledge (de Silva et al., 2025), and environmental consciousness (Sheikh et al., 2025). Therefore, the role of anthropomorphized green brands in the consumption context may be different from that of general brands. Is there a difference in the effect of different types of green brand anthropomorphism on the improvement of purchasing intentions? What is the path through which it affects purchasing intentions? Will this effect change when facing different product attributes? These questions still lack in-depth discussion.

Based on this, we fill the research gap in the following ways. First, distinguishing between competence-oriented and warmth-oriented green brand anthropomorphism and directly comparing their effects. Second, examining the mediating mechanism of psychological distance. Third, establishing product attributes (functional vs. hedonic) as moderating variables affecting the effectiveness of anthropomorphism types. Specifically, this work takes the Stereotype Content Model (SCM), Construal Level Theory (CLT), and Stimulus–Organism–Response Model (S-O-R Model) as the theoretical basis, draws on the research results of Gong et al. (2021) and X. Zhang et al. (2024), divides the types of green brand anthropomorphism into two different categories, competence-oriented and warmth-oriented, constructs an integrated model on the impact of green brand anthropomorphism on consumers’ purchasing intentions, and designs a between-subject experiment to carry out the research. In this study, competence-oriented focuses on the functional advantages of the brand, such as professional technology and environmental protection efficiency; warmth-oriented focuses on the emotional characteristics of the brand, such as emotional care and value resonance. We believe that anthropomorphism can improve consumers’ green product purchase intention more than non-anthropomorphism, and the effect of different types of green brand anthropomorphism on green product purchase intention is different. Specifically, warmth-oriented anthropomorphism can improve consumers’ green purchasing intentions more than competence-oriented anthropomorphism, because the “Primacy-of-Warmth Effect” makes consumers more easily persuaded by information with warm emotions (El Hedhli et al., 2023). In addition, we further speculate that psychological distance plays a significant mediating role in this process, while product attributes play a moderating role. When the selected product has functional attributes, competence-oriented anthropomorphism can better improve the green product purchase intention; when the selected product has hedonic attributes, warmth-oriented anthropomorphism is more effective in improving the purchase intention.

This work offered several theoretical and practical implications. In terms of theoretical contributions, first of all, this study expands the research limitations of “green brand anthropomorphism or not” in the existing research. By distinguishing competence-oriented and warmth-oriented anthropomorphism, it focuses on the impact effect of type differences, enriches the research in the intersection field of green consumption and brand anthropomorphism, and verifies the applicability and effectiveness of the “Primacy-of-Warmth Effect” (Abele & Wojciszke, 2007) in the influence of anthropomorphism on green consumption behavior, providing a more detailed theoretical perspective for the research on green consumption. Secondly, this study introduces psychological distance into the analysis framework of the anthropomorphism influence mechanism, reveals the mediating role of psychological distance as a key variable in consumers’ green decision-making, further explains the internal mechanism of green brand anthropomorphism, and expands the application boundary of Construal Level Theory (CLT) and the Stimulus–Organism–Response Model (S-O-R Model) in the field of green consumption. Third, this study examines the moderating effect of product attributes and proposes that the effectiveness of green brand anthropomorphism is dependent on product attributes, which provides important boundary condition evidence for anthropomorphism research. In terms of practical implications, this study can provide guidance for green brands to accurately select anthropomorphism strategies. Enterprises can be more targeted when shaping anthropomorphized green brand images, according to the findings of the study. By matching product attributes with anthropomorphism features, the efficiency of green concept communication can be improved, and consumers’ willingness to buy can be enhanced. At the same time, by shortening the psychological distance, the abstractness and distance of green value can be weakened so as to improve the emotional appeal and persuasion effect of green marketing activities and promote the sustainable development of the green consumption market.

## 2. Theoretical Framework and Research Hypothesis

### 2.1. Green Brand Anthropomorphism and Purchasing Intentions

Green brands have gradually developed stable values and communication norms centered on environmental responsibility and sustainable value through long-term corporate operations, establishing a distinctive brand culture (Lyeonov et al., 2021; Vysotska & Vysotskyi, 2022). The core lies in elevating “sustainability commitments” from functional selling points to normative brand identities and action guidelines (Blanco-Moreno et al., 2025; Suhan et al., 2022). From a behavioral science perspective, green brands can promote sustainable behaviors not by directly altering environmental performance through brand culture itself, but by shaping consumers’ trust, sense of responsibility, and self-relevance toward the brand. This enhances the probability of eco-friendly choices in purchasing situations (J. Lin et al., 2021). These micro-decisions, when accumulated over time, indirectly contribute to environmental sustainability outcomes. Therefore, this study posits that green brand anthropomorphism influences sustainable behavior by affecting consumers’ green purchasing decisions and pro-environmental choice tendencies. The Stereotype Content Model (SCM) provides a crucial theoretical framework for this research.

The concept of the Stereotype Content Model (SCM) comes from Judge’s investigation in the field of psychology at the end of the last century. The investigation shows that people often use words such as “friendly”, “warm”, “smart” and “professional” when evaluating others or specific things. Based on Judge’s investigation, Fiske et al. (2002) proposed the Stereotype Content Model, which holds that people’s perception of other individuals or groups can be summarized into two core dimensions. One is the evaluation focusing on competence characteristics, such as professionalism and intelligence, and the other is the evaluation focusing on personality characteristics, such as enthusiasm and friendliness. Later, Cuddy et al. (2008) pointed out that competence is the perception and evaluation of the level of effectiveness that others have in achieving their goals, and its scope covers technology, creativity, etc.; while warmth is a more perceptual cognition of others’ intentions, including morality, sincerity, etc. The Stereotype Content Model explains that when the information obtained by an individual is insufficient or unclear, the information is usually classified and summarized into the dimensions of competence and warmth so as to improve the efficiency of decision-making. Similar to the process of efficient decision-making, consumers will also form a perception of the brand on these two basic dimensions. D. A. Aaker (1997) proposed in the study of consumer brand perception that consumers would give the brand human characteristics when evaluating brand characteristics, and these characteristics are consistent with the competence and warmth dimensions in the Stereotype Content Model. Specifically, consumers can feel the competence and warmth of the brand at the same time when shopping. The brand with a high technical level or good product efficacy will be considered as high-competence, while the brand with enthusiastic and friendly advertising or a cute and beautiful product appearance design will be considered as high-warmth (J. L. Aaker et al., 2012). Based on this, this study divides the types of green brand anthropomorphism into two different categories: competence-oriented and warmth-oriented.

In this work, competence-oriented anthropomorphism refers to the brand’s use of anthropomorphism to highlight its own capabilities and make functional and technical commitments to consumers, while warmth-oriented anthropomorphism refers to the brand’s creation of a warm, friendly, and enthusiastic image, focusing on emotional connections with consumers. Competence-oriented anthropomorphism makes consumers believe that a brand is reliable and prompts consumers to fully understand the performance and quality of products, which is conducive to meeting consumers’ functional needs for products, thereby increasing their willingness to buy (Andrei et al., 2017). On the other hand, warmth-oriented anthropomorphism makes consumers feel that the brand is warm, which can make the brand promotion effect and the brand reputation better (F. Liu et al., 2022), and is also an important factor in improving consumers’ willingness to buy. It can be seen that compared with non-anthropomorphism, both competence-oriented and warmth-oriented anthropomorphism can often better convey the image and proposition of green brands to consumers in a positive, active, and vivid way; expand the influence of their brand promotion; and make consumers more clearly aware of the brand’s sustainable development concept and environmental protection responsibility, thereby increasing consumers’ brand preference and improving their willingness to purchase and repurchase (Sehgal et al., 2023).

In the communication of green brands, anthropomorphism through images and language can make consumers feel close. In this process, consumers will have the perception and cognition of the brand as a “person” and often form two different impressions: “warmth-oriented” with high emotional temperature and “competence-oriented” with strong professional skills (Kervyn et al., 2012). This perception and judgment will significantly affect consumers’ value choices and purchasing behavior (R. Huang & Ha, 2020). Some studies have pointed out that the warmth characteristics displayed by brands are more easily recognized by consumers than competence characteristics (Kolbl et al., 2020), which in turn has a greater impact on consumer behavior. El Hedhli et al. (2023) focused on anthropomorphized virtual influencers and found that consumers were more willing to accept product recommendations from warm anthropomorphized virtual influencers than from competent ones. This is because warmth-oriental is more in line with the characteristics and impressions of “people” in the process of anthropomorphism; the “Primacy-of-Warmth Effect” is stimulated; and information with warm emotions has higher persuasive effectiveness. Compared with competence-oriented anthropomorphism, it is easier to promote individual emotional participation and resonance (Tian et al., 2023), thus generating a more positive attitude toward the brand and improving purchasing intentions. Therefore, we believe that compared with competence-oriented anthropomorphism, warmth-oriented anthropomorphism can establish a friendly and emotional brand image through more enthusiastic, warm, and sincere abstract expressions; meet consumers’ cognitive and emotional needs; and is more likely to gain consumers’ favor than competence-oriented anthropomorphism. This makes consumers perceive that a green brand is not a concept in the void but an environmental protection partner who can advocate a low-carbon and beautiful life with them, which is more conducive to making consumers feel close and thus significantly improving their purchasing intentions. Based on the above analysis, this study proposes the following hypotheses:

**H1.**  *Compared to non-anthropomorphism, green brand anthropomorphism is more effective at boosting consumers’ purchasing intentions. Specifically,*

**H1a.**  *Compared to non-anthropomorphism, competence-oriented anthropomorphism is more effective at boosting consumers’ purchasing intentions.*

**H1b.**  *Compared to non-anthropomorphism, warmth-oriented anthropomorphism is more effective at boosting consumers’ purchasing intentions.*

**H2.**  *Different types of anthropomorphism show significant differences in the effect of promoting purchasing intentions. Specifically, warmth-oriented anthropomorphism is more effective than competence-oriented anthropomorphism.*

### 2.2. Mediating Role of Psychological Distance

Psychological distance is the core construct of Construal Level Theory (CLT), which refers to the subjective degree of alienation or proximity between an individual and the target object (including brands, products, events, etc.) perceived at the cognitive level (Trope & Liberman, 2010). The core idea of this theory is that psychological distance is systematically related to the way individuals represent information. That is, the closer the psychological distance is, the more likely individuals are to use low-level construal to process information in a concrete and detailed way; the farther the psychological distance is, the more likely they are to use high-level construal to represent information in an abstract and essential way (Liberman et al., 2007). Psychological distance includes four independent and mutually convertible dimensions: temporal, spatial, social and possibility. Its core characteristics are subjectivity and dynamics. The same objective object may form different distance perceptions in the cognition of different individuals, and this perception will be actively adjusted by external stimulus factors (Trope & Liberman, 2003).

In the field of consumer behavior research, psychological distance is regarded as a key cognitive link connecting brand stimuli and consumer decisions. SOR theory explains the psychological reactions of individuals after receiving external environmental stimuli, in which the internal cognition or emotion is activated, thus promoting the formation of specific motivations and finally manifesting in explicit behavioral reactions (Guo et al., 2021). This external stimulus usually acts on people’s psychology, which in turn causes behavioral responses. In this process, psychological responses connect external stimuli and individual behavior (Nieves-Pavon et al., 2023). Existing studies have confirmed that psychological distance indirectly affects consumers’ attitudes and behavioral tendencies by influencing the fluency of information processing and the intensity of emotional connection (Liberman & Trope, 2008). When the psychological distance between consumers and brands is relatively close, brand information is more easily incorporated into the individual’s self-cognitive framework, stimulating a stronger willingness to pay attention and emotional resonance, thereby increasing the acceptance and trust of the brand (Aggarwal & McGill, 2007). This mechanism is particularly prominent in the interaction between brands and consumers and has become an important theoretical perspective for explaining the differences in the effect of brand communication. It also provides theoretical support for the research on the influence of green brand anthropomorphism on consumer behavior.

Brand anthropomorphism breaks the traditional “human–object” interaction framework by giving brands human characteristics, emotions, or behavior patterns and builds a simulated “human–human” interaction situation (Touré-Tillery & McGill, 2015). This quasi-interpersonal interaction model mainly achieves its effect on psychological distance through three core paths. First, anthropomorphism activates consumers’ human cognitive representation and shortens social distance perception. Brand anthropomorphism makes the brand have human social attributes through language style (such as first-person expression), emotional expression (such as empathetic communication), and behavioral characteristics (such as interactive response), so as to reduce consumers’ “non-human” perception of the brand (Rauschnabel & Ahuvia, 2014). When a brand is anthropomorphized, consumers will automatically activate the cognitive script of interpersonal interaction in the brain and regard the brand as a social object that can be communicated with rather than a simple product carrier. This cognitive transformation significantly shortens the social distance between the brand and consumers (Aggarwal & McGill, 2007). Second, anthropomorphism improves the fluency of information processing and weakens the cognitive obstacles caused by psychological distance. The core value proposition of green brands (such as environmental protection attributes and sustainable development concepts) often involves professional knowledge and abstract concepts, which can easily cause cognitive barriers and psychological alienation among consumers (Ricci et al., 2018). Brand anthropomorphism, on the other hand, reduces the difficulty of information decoding and improves cognitive fluency through concrete expression and interactive communication (He et al., 2020). For example, anthropomorphized brands use first-person statements such as “I promise to use biodegradable materials” to transform abstract environmental protection commitments into specific interpersonal commitments, making it easier for consumers to understand and accept brand information. Finally, anthropomorphism promotes the formation of emotional connection and strengthens the effect of shortening psychological distance. Emotional resonance is the key driving force for shortening psychological distance, and brand anthropomorphism just provides a carrier for emotional connection (M. Zhang et al., 2020). Brand anthropomorphism can express human emotions such as joy and responsibility so that consumers can produce an empathy experience in the interaction. This emotional resonance will enhance consumers’ familiarity and affinity for the brand, thus further shortening the psychological distance (White & Peloza, 2009). In the context of green consumption, this emotional connection is particularly important, because consumers’ green purchasing behavior often contains altruistic motives, and the emotional expression of brand anthropomorphism can better stimulate consumers’ pro-social emotions and strengthen psychological closeness.

The value proposition of green brands determines that it is easier to produce a natural psychological distance between them and consumers. On the one hand, the environmental performance of green products is difficult to verify through intuitive experience, and consumers have uncertainty about the perception of their actual environmental protection effects. This information asymmetry will exacerbate psychological alienation (Gleim et al., 2013); on the other hand, the long-term benefits of green consumption conflict with the immediate interests of consumers, which easily makes consumers feel a sense of distance of “it’s none of my business” and reduces their willingness to buy (Sun et al., 2018). Green brand anthropomorphism, on the other hand, can break this dilemma by shortening the psychological distance through the above mechanism. On the one hand, green brand anthropomorphism shortens psychological distance, making consumers more willing to actively process the brand’s environmental information. According to CLT, when psychological distance is short, consumers tend to use concrete processing and pay more attention to the specific details and feasibility of brand information (Trope & Liberman, 2010), which helps consumers to deeply understand the environmental value and use scenarios of green products, thereby improving the acceptance of brand information (Cryder et al., 2013). On the contrary, if the psychological distance is far, consumers tend to process information abstractly, easily ignoring key information and reducing the efficiency of information acceptance. Therefore, green brand anthropomorphism creates conditions for consumers to deeply process environmental information by shortening the psychological distance, thus laying the foundation for the improvement of purchase intention. On the other hand, green consumption behavior is not only driven by rational cognition but also deeply influenced by emotional factors (Hartmann & Apaolaza-Ibáñez, 2012). Therefore, shortening psychological distance can enhance consumers’ emotional identification with green brands so that consumers can internalize the brand’s environmental protection goals into their own value pursuit (Wang et al., 2017). The emotional connection established by brand anthropomorphism through quasi-interpersonal interaction will be strengthened with the shortening of psychological distance, making consumers trust and like the brand, and this emotional identity will be directly transformed into purchase intention (White et al., 2019).

Specifically, competence-oriented anthropomorphism takes functional display and professional ability as the core and mainly shortens the psychological distance by improving the credibility of information. These brands emphasize ability characteristics such as environmental protection technology and certification qualifications to establish consumers’ trust in the environmental protection effectiveness of the brand (Gupta et al., 2019). Trust, as the basis of interpersonal interaction, can effectively reduce consumers’ cognitive barriers to brands and shorten psychological distance. When green brands can clearly demonstrate their environmental protection capabilities, consumers’ psychological alienation from the brand will be significantly reduced (Gleim et al., 2013). Warmth-oriented anthropomorphism, with emotional expression and interpersonal care as its core, is better at shortening psychological distance through emotional resonance. This type of brand anthropomorphism simulates intimate interpersonal interactions by transmitting emotional signals such as warmth and empathy, making consumers feel psychologically close (Rauschnabel & Ahuvia, 2014). This sense of closeness will further enhance the trust and purchase intention of the brand (White & Peloza, 2009). In the context of green consumption, warmth-oriented anthropomorphism can directly respond to consumers’ altruistic motives and emotional needs. Through emotional expression, it binds consumers to the brand’s environmental protection goals, significantly shortens the psychological distance, strengthens emotional identity, and then deeply stimulates consumers’ willingness to purchase green products.

From the behavioral mechanism perspective, psychological distance connects brand stimuli to green decision-making primarily through three pathways. First, as psychological distance decreases, consumers tend to process information in more concrete and detailed ways. This facilitates understanding of green products’ usage scenarios and environmental value, leading to stronger feasibility judgments (Luan et al., 2023). Second, as psychological distance decreases, green brands and their environmental goals become more readily integrated into consumers’ self-relevant frameworks. This enhances perceived value congruence and sense of responsibility, strengthening consumer-brand interaction (Kull et al., 2021) and preventing green choices from being dismissed as abstract, “not my problem” issues. Third, reduced psychological distance enhances emotional closeness and trust while diminishing skepticism toward green promises and psychological barriers stemming from information asymmetry (Gerrath et al., 2024). Based on these mechanisms, the mediating pathway through which green brand anthropomorphism increases green purchasing intentions by shortening psychological distance possesses a clear behavioral science foundation. Based on the above analysis, this study proposes the following hypotheses:

**H3.**  *Psychological distance mediates the effect of green brand anthropomorphism on consumers’ purchasing intentions.*

### 2.3. Moderating Role of Product Attributes

Product attributes, as an important basis for consumers to perceive and evaluate products, are widely divided into two dimensions: functional and hedonic attributes (Y. Zhang & Wang, 2023). Functional attributes are also referred to as utilitarian in some studies (Choi et al., 2020). Functional attributes are characterized by instrumentality and practicality and are designed to help consumers solve specific problems, complete specific tasks, or achieve utilitarian goals. Their value is mainly reflected in objective indicators such as efficiency, reliability, and functionality (Botti & McGill, 2011). For example, the core basis for consumers’ purchase decisions of green products such as energy-saving household appliances and environmentally friendly cleaning products is their environmental protection efficiency, durability, and practical value, and they pay attention to whether the products can effectively meet the needs of environmentally friendly use. In the consumption process of such products, consumers usually need to fully understand the functional attributes, technical information, etc., of the products and then make a choice after comparing and analyzing the product information obtained (Kivetz & Zheng, 2017). They are more inclined to conduct rational analysis, pay attention to the matching degree between product attributes and their actual needs, and emphasize the functional return of decision-making. Hedonic attributes, on the other hand, take emotional experience and sensory pleasure as the core value, aiming to meet consumers’ emotional needs, esthetic preferences, or entertainment experience, and its value comes from subjective feelings such as happiness, satisfaction, and self-expression brought by the use of products (Shao & Li, 2021). Green hedonic products, such as organic skin care products and environmentally friendly designed home decorations, are purchased by consumers who not only pay attention to their environmental attributes but also attach importance to the psychological benefits, such as emotional resonance, esthetic experience, and social recognition, brought by the products (Qin et al., 2025). In the consumption decision-making of such products, consumers’ emotional demands dominate, and they are more likely to be affected by factors such as emotional connection and brand image and pursue the fit between products and their own emotional needs and lifestyles (F. Liu et al., 2022). There are significant differences in consumer cognition caused by products with different attributes. Functional products activate consumers’ “instrumental cognition”, with the core focus on the effectiveness, reliability, and practicality of the products, while hedonic products activate “emotional cognition”, with the core focus on the emotional value, experience, and pleasure of the products (Y. Zhang & Wang, 2023). This cognitive difference provides an important prerequisite for the matching effect of brand anthropomorphism types and product attributes and also constitutes the cognitive basis of its influence.

Schema congruity theory provides theoretical support for the mechanism of product attributes affecting purchase intention. The theory holds that consumers’ evaluation of products depends on the degree of consistency between product information and their own existing cognition. When external stimuli (such as brand anthropomorphism images) are in line with consumers’ cognitive schema, consumers can process information more smoothly and generate more positive attitudes and behavioral tendencies (Aggarwal & McGill, 2007; Trzebiński et al., 2023). In the context of green brand anthropomorphism, anthropomorphism types (competence and warmth) are important brand information, and the degree of consistency between them and the consumer’s cognitive schema triggered by product attributes directly affects the effectiveness of the anthropomorphism strategy.

For functional green products, their environmental protection effectiveness often has a certain degree of professionalism and concealment, and it is difficult for consumers to directly judge their actual effects. The professional ability signal transmitted by competence-oriented anthropomorphism can concretize these abstract professional advantages into human characteristics that are easily perceived by consumers (Kervyn et al., 2012), which can effectively reduce consumers’ perception of information asymmetry and improve product attractiveness (Kolbl et al., 2020). The cognitive schema of consumers is centered on “instrumentality” and “effectiveness”. At this time, competence-oriented anthropomorphism images that are consistent with this schema are more likely to be recognized by consumers. This image emphasizes the brand’s professional capabilities, high-efficiency performance, and reliable quality (Kervyn et al., 2012). The information it conveys, such as “professionally solving environmental problems” and “efficiently realizing environmental protection functions,” is highly consistent with the instrumental cognitive schema of functional products and can accurately respond to consumers’ rational decision-making demands (F. Liu et al., 2022), forming a significant matching advantage, strengthening consumers’ trust in the environmental protection effectiveness and practical value of products (Kolbl et al., 2020), and then improving the willingness to buy. On the contrary, information such as emotional care and friendly interaction emphasized by warmth-oriented anthropomorphism is less consistent with the cognitive schema of functional products, and it is difficult to meet the core needs of consumers for product effectiveness, and its positive effect will be suppressed (Y. Zhang & Wang, 2023).

For hedonic green products, their core value lies in providing consumers with emotional experience and psychological satisfaction, and consumers’ purchase decisions are more driven by factors such as emotional appeal, esthetic preference, and social identity (Shao & Li, 2021). Warmth-oriented anthropomorphism highlights the brand’s friendly attitude, emotional care, and social responsibility. The information it conveys, such as “paying attention to consumers’ emotional needs” and “practicing an environmentally friendly lifestyle with consumers,” is highly matched with the emotional cognitive schema of hedonic attributes, which can enhance the emotional connection between consumers and the brand, allowing consumers to obtain emotional satisfaction while strengthening their recognition of the brand’s environmental protection concept, thereby increasing their willingness to purchase. Existing studies have confirmed that anthropomorphism can improve consumers’ perceived suitability of hedonic attribute appeals (Trzebiński et al., 2023) and change consumers’ understanding and processing of product attributes (Wan et al., 2017), while warmth-oriented anthropomorphism further strengthens this emotional fit, making consumers more willing to accept and purchase products (K. Kim et al., 2019). In addition, the experiential value of hedonic products not only comes from the product itself but also from the interaction between the brand and consumers, and warmth-oriented anthropomorphism can make the interaction between the brand and consumers more humanized and emotional (Aggarwal & McGill, 2012). For example, green home furnishing brands interact with consumers through warmth-oriented anthropomorphism images to convey the environmental protection design concept and emotional value of products, which can enable consumers to obtain a richer emotional experience in the process of purchasing and using products and then enhance the overall attractiveness of products. The information emphasized by competence-oriented anthropomorphism, such as professional efficacy, has a low degree of fit with the emotional appeal of hedonic attributes, and it is difficult to trigger consumers’ emotional resonance, and its promotion effect is relatively limited (Trzebiński et al., 2023).

In addition, anthropomorphism, as an effective brand communication strategy, can reduce the psychological distance between consumers and brands by giving brands human characteristics (Aggarwal & McGill, 2012; Stinnett et al., 2013). Product attributes, as an important dimension for consumers to perceive products, can regulate the relationship between brand anthropomorphism and psychological distance; that is, the impact of competence- and warmth-oriented anthropomorphism on psychological distance is different under different attributes. For functional green products, the psychological distance between consumers and brands mainly stems from distrust of product efficacy and cognitive barriers, while competence-oriented anthropomorphism can effectively reduce this cognitive barrier by transmitting professional and reliable signals (Qin et al., 2025). This anthropomorphism makes consumers believe that the brand has the professional ability to solve practical environmental problems, and this sense of trust can narrow the psychological distance between consumers and the brand, making consumers more willing to accept and identify with the brand (Kolbl et al., 2020). For example, functional green home appliance brands use competence-oriented anthropomorphism images to show consumers their professionalism and reliability in energy-saving technology, which can make consumers feel the fit between the brand and their actual needs and then reduce the psychological distance. For hedonic green products, the psychological distance between consumers and brands mainly stems from the lack of emotional connection, while warmth-oriented anthropomorphism can effectively reduce this emotional gap by building an emotional bridge (F. Liu et al., 2022). Warmth-oriented anthropomorphism gives the brand friendly and caring human characteristics, allowing consumers to feel the emotional temperature of the brand, and this emotional resonance can narrow the psychological distance between consumers and the brand (Aggarwal & McGill, 2012). For example, the hedonic organic skin care brand conveys the emotional value of caring for the skin and the environment to consumers through warmth-oriented anthropomorphism images, which can make consumers feel the degree of fit between the brand and their own emotional needs, and then generate a sense of intimacy. In addition, the Schema Congruity Theory can also explain the moderating effect of product attributes on the relationship between brand anthropomorphism and psychological distance. When the cognitive schema triggered by brand anthropomorphism is consistent with that triggered by product attributes, consumers can process brand information more smoothly, enhance their understanding and identification of the brand, and then more effectively reduce psychological distance (Aggarwal & McGill, 2007). The schema consistency between functional attributes and competence-oriented anthropomorphism and the schema consistency between hedonic attributes and warmth-oriented anthropomorphism can both improve the fluency of consumers’ information processing, strengthen brand identity, and thus significantly reduce psychological distance. On the contrary, inconsistent combinations will increase the difficulty of information processing, weaken brand identity, and have a relatively limited impact on psychological distance. Based on the above analysis, this study proposes the following hypotheses:

**H4.**  *Product attributes play a moderating role in the effect of green brand anthropomorphism on consumers’ purchasing intentions. Specifically,*

**H4a.**  *For functional attributes, competence-oriented anthropomorphism can more effectively boost purchasing intentions.*

**H4b.**  *For hedonic attributes, warmth-oriented anthropomorphism can more effectively boost consumer purchasing intent.*

**H5.**  *Product attributes play a moderating role in the effect of green brand anthropomorphism on psychological distance.*

**Figure 1:**
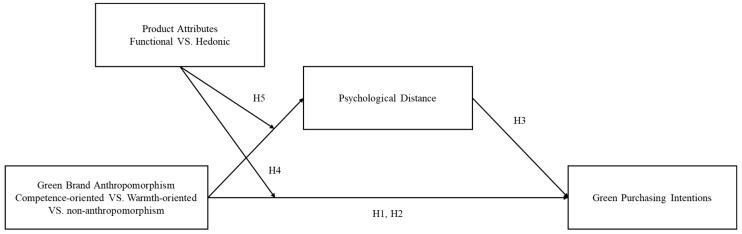
Research Model.

In order to intuitively show the relationship between the variables, we drew the research model as shown in Figure 1. We also present the correspondence between each research hypothesis and the corresponding studies in Table 1 to clarify which study tested each hypothesis.

## 3. Study 1

### 3.1. Pilot Test: Green Brand Anthropomorphism Types Manipulation

#### 3.1.1. Pilot Test 1: Word Validation

The purpose of Pilot Test 1 is to verify that the words or phrases used in the copy for “competence-oriented/warmth-oriented” are indeed perceived as competence or warmth. Drawing from online advertisements featuring “competence” and “warmth” expressions, we compiled representative words/phrases into a “competence lexicon” (e.g., “high efficiency/energy-saving/adaptive operation/innovative foaming technology”) and a “warmth lexicon” (e.g., “care for/walk alongside/love for life”) (see the Supplementary Materials).

We recruited 76 participants (N_female_ = 60%) via Credamo (a professional survey platform). Participants were randomly assigned to different orientation word groups and rated candidate words or phrases using a 7-point scale (1 = Strongly Disagree, 7 = Strongly Agree). Competence perception was measured through the following items: (1) “This word/phrase feels competent”; (2) “This word/phrase conveys capability and reliability”. Warmth perception was measured by (1) “This word/phrase feels warm”; (2) “This word/phrase conveys care and thoughtfulness”.

Results indicate that most words in the competence lexicon scored significantly higher on competence perception than warmth perception (M_competence_ = 5.89, M_warmth_ = 4.86, t = 4.93, *p* = 0.000); conversely, most words in the warmth lexicon scored significantly higher on warmth perception than competence perception (M_competence_ = 5.54, M_warmth_ = 6.21, t = −3.04, *p* = 0.004). Based on these findings, we selected words with significantly higher competence perception scores as core cues for competence-oriented copywriting, while words with significantly higher warmth perception scores were chosen as core cues for warmth-oriented copywriting. This provides a basis for constructing stimulus materials. Thus, Pilot Test 1 demonstrated that textual cues used to distinguish competence-oriented and warmth-oriented anthropomorphism possess good manipulation validity.

#### 3.1.2. Pilot Test 2: Image Validation

After validating the textual cues, we proceeded to conduct Pilot Test 2 to verify the image cues used in Study 1 to express competence-oriented and warmth-oriented anthropomorphism. The key objective of this pilot test was to examine whether the images alone (without presenting any textual information) could elicit distinct perceptions of competence and warmth among participants, thereby ruling out the possibility that textual content drove these differences. For Pilot Test 2, we selected refrigerators as stimuli for material design, presenting competence-oriented and warmth-oriented anthropomorphism images (see the Supplementary Materials).

We recruited 75 participants via Credamo for evaluation (N_female_ = 65%). Participants were randomly assigned to different anthropomorphism image groups and reported their perceptions of the images. We employed a 7-point scale for scoring. Competence perception was measured via the following items: (1) “This image feels very competent”; (2) “This image conveys capability and reliability.” Warmth perception was measured via (1) “This image feels very warm”; (2) “This image conveys care and thoughtfulness.”

Results indicated that competence images scored significantly higher on competence perception than warmth perception (M_competence_ = 5.86, M_warmth_ = 5.28, t = 3.44, *p* = 0.002), while warmth images scored significantly higher on warmth perception than competence perception (M_competence_ = 4.90, M_warmth_ = 5.90, t = −4.19, *p* = 0.000), confirming the validity of the anthropomorphism manipulation premise. This indicates that the selected competence and warmth images can reliably distinguish competence and warmth perceptions without relying on textual information.

#### 3.1.3. Pilot Test 3: Same-Copy and Different-Images Validation

Pilot Test 3 aims to validate the independent effect of image cues through a “same-copy & different-images” approach. Specifically, we selected refrigerators as stimuli for material design, using identical brand copy across both experimental conditions (avoiding prominent competence/warmth lexical cues to ensure textual neutrality) but pairing them with competence- and warmth-oriented anthropomorphic images (see the Supplementary Materials). This design maximally ruled out alternative explanations where “differences in the competence/warmth copy itself (e.g., being more interesting or emotionally appealing) caused the effect,” thereby testing whether image cues alone were sufficient to drive participants’ perceptual differences in anthropomorphism types.

We recruited 69 participants (N_female_ = 65%) via Credamo and randomly assigned them to two groups (competence image vs. warmth image, each paired with an identical copy). After reading the same copy and viewing the corresponding image, participants completed manipulation check items. The manipulation check included competence and warmth perception measures (identical to Pilot Test 2).

Results revealed that even with identical copy, simply replacing the image significantly differentiated participants’ competence and warmth perceptions. The competence image group exhibited significantly higher competence perception than warmth perception (M_competence_ = 5.81, M_warmth_ = 5.32, t = 2.76, *p* = 0.009), while the warmth perception in the warmth image group was significantly higher than competence perception (M_competence_ = 5.25, M_warmth_ = 5.96, t = −3.77, *p* = 0.001). Therefore, Pilot Test 3 further supports that even under identical copy conditions, image cues themselves can drive distinct perceptions of competence-oriented and warmth-oriented anthropomorphism, effectively ruling out the alternative explanation that “copy differences caused the effect.”

#### 3.1.4. Pilot Test 4: Different-Copy and Different-Images Validation

The purpose of pilot test 4 is to manipulate and test the presentation content of different green brand anthropomorphism types. Study 1 selected refrigerators with strong green attributes as stimuli and adopted the virtual brand “EcoFreeze”. Referring to the combination of pictures and texts in existing studies (Li et al., 2025), we used Photoshop CS6 software to design brand anthropomorphism images and used first-person dialogs and daily greetings to imitate the characteristics of human speech (see the Supplementary Materials). We used Question Star (a professional online survey platform) to distribute questionnaires and recruited a total of 42 participants. The pilot test required participants to judge the degree of anthropomorphism of green brands and to score on a 7-level scale (1 = totally non-anthropomorphism, 7 = totally anthropomorphism) to test whether the display content of refrigerators could correctly show the difference between anthropomorphism and non-anthropomorphism. We used one-way ANOVA analysis, and the results showed that the score of non-anthropomorphism was significantly lower than that of competence-oriented anthropomorphism and warmth-oriented anthropomorphism (M_non-anthropomorphism_ = 2.24, M_competence_ = 5.69, *p* = 0.000; M_non-anthropomorphism_ = 2.24, M_warmth_ = 5.83, *p* = 0.000). Further post hoc multiple comparisons revealed that there was no significant difference in the mean score of anthropomorphism between the competence-oriented and warmth-oriented groups (*p* = 0.577). Therefore, the experimental materials of the refrigerator can effectively distinguish whether it is anthropomorphism or not and can be used for formal experiments.

Furthermore, we tested whether the experimental stimulus materials of refrigerators could correctly show the differences between different green brand anthropomorphism types, that is, whether the participants could judge and identify the two different types of competence-oriented and warmth-oriented. We asked participants to rate the anthropomorphism types on a 7-point scale (1 = competence, 7 = warmth). The results of the paired-samples *t*-test showed that the mean of the warmth-oriented display content of the refrigerator was significantly higher than that of the competence-oriented display content (M_competence_ = 2.98, M_warmth_ = 5.26, *p* = 0.000). Therefore, we believe that the design of the experimental materials is reasonable and can be used for formal experiments.

### 3.2. Experimental Procedure

The primary objective of Study 1 is to verify whether green brand anthropomorphism, compared to non-anthropomorphism, can effectively enhance purchasing intentions. It also examines whether different anthropomorphism types (competence-oriented vs. warmth-oriented) lead to distinct green purchasing intentions, and tests the role of psychological distance as a behavioral mechanism in this process, testing H1, H2, H3.

Study 1 conducted a single-factor three-level (competence-oriented vs. warmth-oriented vs. non-anthropomorphism) between-subjects experiment and selected household smart refrigerators as experimental stimuli. A total of 223 participants were recruited through the professional questionnaire platform Credamo (N_female_ = 53%, excluding samples with careless answers). Demographic information was shown in Table 2. Study 2 randomly assigned participants to three experimental groups. The participants were told to “ Imagine you’re shopping for a refrigerator. While browsing an online shopping site, you come across the following refrigerator brand advertisement.” Subsequently, they read the corresponding stimulus materials. After guiding the participants to watch and read the experimental materials, they were asked to answer the 7-point scale of purchasing intentions and psychological distance (1 = strongly disagree, 7 = strongly agree) according to their actual feelings. Given that refrigerators are high-priced, low-frequency durable goods, the purchasing intentions examined in this study represent choices made in an imagined refrigerator-shopping scenario. Therefore, the following scale measures participants’ propensity to consider the brand in specific need situations following advertising exposure. The scale of the dependent variable, consumers’ purchasing intentions, referred to Li et al. (2025), including (1) “I would consider buying this brand’s products”; (2) “I am very likely to buy this product”; (3) “I think this product is worth buying”; (4) “I am willing to recommend this product to others if they need it”. The psychological distance scale was based on Darke et al. (2016) and Lim et al. (2012) to adapt to the research needs of Study 1, including: (1) “I feel that the brand can give me a sense of intimacy”; (2) “I feel that the image has brought me closer to the brand”; (3) “I feel that the image has reduced my strangeness to the brand”; (4) “I feel that the image can make me feel a sense of belonging and identity”. After completing the above tasks, participants were also required to answer the manipulation items about green brand anthropomorphism, including competence-oriented and warmth-oriented dimensions. The manipulation test items were measured by Likert 7-point scales. Finally, participants were asked to provide basic demographic information.

### 3.3. Results

#### 3.3.1. Reliability and Validity Analysis

Study 1 used SPSS 25.0 to calculate the Cronbach’s Alpha value of the scale. It was found that the Cronbach’s Alpha values of psychological distance and purchasing intentions were 0.920 and 0.877, respectively, at the 95% confidence interval level, and the value of the total scale was 0.911, which were all greater than 0.8, indicating that the reliability of the questionnaire scale in this study was good. The structural validity test was conducted using AMOS 24.0. The results are shown in Table 3. The standardized factor loading values of all items in this study were above 0.5, the AVE value of psychological distance reached 0.744, and the AVE value of purchasing intentions was 0.649, both of which were greater than 0.5. The CR values were 0.921 and 0.880, respectively, both of which were greater than 0.7. Thus, the questionnaire scale of this study has good convergent validity.

The results of the discriminant validity test of the scale are shown in Table 4 below. The square root of the AVE value of each construct is significantly larger than the correlation coefficient between the constructs, and it can be determined that the discriminant validity of the questionnaire scale in this study is good. It can be seen that the validity of the questionnaire scale in this study is good.

#### 3.3.2. Test of Main Effects

First, Study 1 conducted a manipulation check. The results of the one-way ANOVA test showed that there was a significant difference in the mean of green brand anthropomorphism among the three experimental groups (M_non-anthropomorphism_ = 2.60, M_competence_ = 5.06, M_warmth_ = 5.36, *p* = 0.000). Further post hoc multiple comparisons revealed that the means of the competence-oriented and warmth-oriented groups were significantly higher than the mean of the non-anthropomorphism group (*p* = 0.000), while the difference between the means of the competence-oriented and warmth-oriented groups was not significant (*p* = 0.359). Thus, it can be proved that the green brand anthropomorphism manipulation is successful. The results of the independent sample *t*-test showed that in the competence-oriented experimental group, the mean value of the participants’ self-reported “competence” was significantly higher (M_competence_ = 5.04, M_warmth_ = 3.35, t = 7.415, *p* = 0.000); on the contrary, in the warmth-oriented experimental group, the mean value of the participants’ self-reported “warmth” was significantly higher (M_competence_ = 3.26, M_warmth_ = 5.32, t = −9.470, *p* = 0.000). Thus, it can be proved that the manipulation of different green brand anthropomorphism is successful.

Next, Study 1 conducted the main effect test. The results of the one-way ANOVA test showed that there was a significant difference in the impact of competence-oriented (M_competence_ = 5.03, SD = 1.71), warmth-oriented (M_warmth_ = 5.92, SD = 0.60), and non-anthropomorphism (M_non-anthropomorphism_ = 3.86, SD = 1.28) on purchasing intentions (*p* = 0.000). Further post hoc multiple comparisons revealed that the mean of the competence-oriented group was significantly higher than that of the non-anthropomorphism group (M_competence_ = 5.03, M_non-anthropomorphism_ = 3.86, *p* = 0.000) in the measurement of the purchasing intentions dimension. Compared with non-anthropomorphism, competence-oriented anthropomorphism can better improve consumers’ purchasing intentions and have a positive impact, and H1a is established. Similarly, in the measurement of purchasing intentions, the mean of the warmth-oriented group was significantly higher than that of the non-anthropomorphism group (M_warmth_ = 5.92, M_non-anthropomorphism_ = 3.86, *p* = 0.000). Therefore, compared with non-anthropomorphism, warmth-oriented anthropomorphism can better improve consumers’ purchasing intentions, and H1b is established. Furthermore, we found that the mean of the warmth-oriented group was significantly higher than that of the competence-oriented group in the measurement of purchasing intentions (M_competence_ = 5.03, M_warmth_ = 5.92, *p* = 0.000), which proved that warmth-oriented anthropomorphism had a more positive impact on purchasing intentions than competence-oriented anthropomorphism, verifying H2.

#### 3.3.3. Test of Mediating Effect

In this study, the PROCESS plug-in in SPSS25.0 was used to test whether psychological distance played a mediating role in the research model. Model 4 was selected for bootstrap analysis, and repeated sampling was set for 5000 times under the 95% confidence level. The test results are shown in Table 5. The total effect value of green brand anthropomorphism on purchasing intentions is 0.894, and the direct effect value is 0.416. The two statistical indicators are significant (*p* < 0.01), and the confidence interval of the total effect is (0.471, 1.316), which does not include 0. The confidence interval of the direct effect is (0.116, 0.716), which does not include 0, and the main effect is verified again. The statistical indicator of the indirect effect of the mediating variable psychological distance is also significant (*p* = 0.007), the confidence interval is (0.167, 0.821), and it does not contain 0, and the effect value is 0.478. Therefore, psychological distance plays a partial mediating role between green brand anthropomorphism and purchasing intentions, which verifies H3.

## 4. Study 2

### 4.1. Pilot Test: Product Attributes Manipulation

The purpose of this pilot test is to determine the practical functional and hedonic products that represent different product attributes and are needed in the experiment of this study. We first selected 11 green products that are common in life and frequently used in relevant studies through focus group discussions, including green laundry detergent, natural juice, energy-saving refrigerators, energy-saving smartwatches, green toilet paper, wheat straw cups, energy-saving game consoles, natural toothpaste, environmentally friendly headphones, environmentally friendly perfume, and new energy vehicles. We recruited 77 participants (N_female_ = 58%) from Question Star (a professional survey platform) to join this pilot test. First, the subjects were asked to judge the green attributes of the products and score them on a 7-point scale (1 = not green at all, 7 = completely green; the higher the score, the stronger the green attributes of the products). The results showed that the average score of the green attributes of the products was above 5 points, except for the average score of the energy-saving smartwatch, which was 4.95. It can be seen that the green products selected in this pre-experiment all have green attributes and meet the conditions required for this research experiment. Next, we asked the participants to judge the product attributes and score them on a 7-point scale (1 = totally functional, 7 = totally hedonic). The lower the score, the stronger the functional attributes; on the contrary, the higher the score, the stronger the hedonic attributes. By sorting the mean scores from high to low, we finally selected the wheat straw water cup (M = 2.47) as the representative of functional attributes and the environmentally friendly perfume (M = 5.84) as the representative of hedonic attributes in the experiment. In Study 2, we used the wheat straw cup and the eco-friendly perfume selected in the pilot test as stimuli. We designed the virtual brand “Wheat” to represent the cup and “NatureScent” to represent the perfume through brainstorming, and designed the corresponding experimental materials (see the Supplementary Materials). The purpose of our pilot test was not merely to select two products, but to identify stimulus materials that best represent “functional” and “hedonic” attributes across multiple candidate green product categories. In the pilot test, participants evaluated the significance of functional, hedonic, and green attributes across candidate categories. Using the criterion of “significant perceived differences in functional/hedonic attributes coupled with clear green attributes,” we ultimately selected the straw water cup and the environmentally friendly perfume as formal experimental stimuli.

We further tested whether the experimental stimulus materials of the water cup and perfume could correctly show the difference between different green brand anthropomorphism. We asked the subjects to score the degree of anthropomorphism of the stimuli on a 7-point scale (1 = totally competence-oriented, 7 = totally warmth-oriented). Paired sample *t*-tests were performed using SPSS 25.0 software. The results showed that the subjects’ scores for wheat straw cups (M_competence_ = 2.79, M_warmth_ = 5.21) and environmentally friendly perfumes (M_competence_ = 2.95, M_warmth_ = 5.40) showed that there were significant differences in their perception and judgment of competence and warmth. Therefore, the design of the experimental materials is reasonable and can be used for formal experiments.

### 4.2. Experimental Procedure

Study 2 aims to further examine the moderating role of product attributes (functional vs. hedonic) based on Study 1, addressing the theoretical question of “under which product contexts do specific anthropomorphism types prove more effective.” It also tests whether psychological distance continues to function within the moderated mediation model and enhances the robustness and generalizability of findings by varying stimulus categories, testing H4, H5.

Study 2 conducted a 2 (green brand anthropomorphism: competence-oriented vs. warmth-oriented) × 2 (product attributes: functional vs. hedonic) two-factor between-subjects experiment and selected wheat straw cups and environmentally friendly perfumes as experimental stimuli. A total of 281 participants were recruited through the professional questionnaire platform Credamo (N_female_ = 57%, excluding samples with careless answers). Demographic information was shown in Table 6. The participants were randomly divided into four groups, named “competence + functional”, “competence + hedonic”, “warmth + functional”, and “warmth + hedonic” groups. The participants were told to “Imagine you’re shopping for a water cup/perfume. While browsing an online shopping site, you come across the following brand advertisement.” Subsequently, they read the corresponding stimulus materials. After guiding the subjects to read the experimental materials, they were required to fill in the questionnaire items according to their actual feelings. Among them, the manipulation items of green brand anthropomorphism and the measurement scales of psychological distance and purchasing intentions are the same as those in Study 1. In addition, we added manipulation items of product attributes. All scales were measured on a seven-point Likert scale, and participants provided basic demographic information at the end.

The rationale for employing two studies lies in Study 1’s focus on examining the core effect and the mediating mechanism of psychological distance, while Study 2 concentrates on testing boundary conditions and cross-stimulus robustness, thereby forming a more comprehensive theoretical evidence chain. The experimental research may have limitations regarding stimulus materials and sample composition. First, the pictorial stimuli represent simulated scenarios and differ from real shopping environments. Second, the sample originates from an online platform, and its demographic structure may not fully represent the general population. To mitigate the impact of these limitations on conclusions, we conducted multiple rounds of pre-tests and manipulation checks during stimulus development. Additionally, in Study 2, we replaced stimuli and brand contexts to test the replicability of findings. Regarding sample composition, we imposed no restrictions on gender, ethnicity, or region during data collection to ensure broad representativeness. Additionally, we implemented measures to guarantee sample independence, including “prohibiting duplicate responses from the same IP address”, “forbidding forwarding of surveys”, and “disallowing copy–paste responses”.

### 4.3. Results

#### 4.3.1. Reliability and Validity Analysis

The Cronbach’s Alpha values of psychological distance and purchasing intentions calculated by SPSS25.0 were 0.858 and 0.931, respectively, and the value of the total scale was 0.876, all of which were greater than 0.8, indicating that the reliability was good. AMOS 24.0 was used to test the structural validity of the questionnaire scale, and the results of the convergent validity analysis are shown in Table 7. The standardized factor loadings of each construct in Study 2 were all greater than 0.7, the AVE value of psychological distance was 0.612, and the AVE value of purchasing intentions was 0.773, both of which were greater than 0.5, and the CR values were 0.862 and 0.931, both of which were greater than 0.7. Therefore, the scale has good convergent validity.

Furthermore, we conducted discriminant validity analysis of the questionnaire scale, and the results are shown in Table 8. The square root of the AVE value of each construct is significantly larger than the correlation coefficient between the constructs, and it can be determined that the discriminant validity of the questionnaire scale in this study has reached a good standard. Therefore, the validity of the questionnaire scale in this study is good.

#### 4.3.2. Test of Main Effects

First, Study 2 conducted a manipulation check. The manipulation items of product attributes adopted the Likert 7-point scale (1 = totally functional, 7 = totally hedonic). The lower the score, the stronger the functional attributes of the product, and the higher the score, the stronger the hedonic attributes of the product. The results of the *t*-test showed that the mean of the functional group was significantly lower than that of the hedonic group (M_functional_ = 2.25, M_hedonic_ = 5.22), and the difference in participants’ perception of different product attributes was significant (*p* = 0.000), which reflected the successful manipulation of product attributes. Next, the manipulation of green brand anthropomorphism was tested. The results showed that in the competence dimension, the mean of the competence-oriented group was significantly higher than that of the warmth-oriented group (M_competence_ = 5.17, M_warmth_ = 3.84, t = 8.74, *p* = 0.000), while in the warmth dimension, the mean of the warmth-oriented group was significantly higher than that of the competence-oriented group (M_competence_ = 3.79, M_warmth_ = 5.19, t = −9.37, *p* = 0.000). Therefore, the manipulation of different green brand anthropomorphism was successful.

Next, Study 2 conducted the main effect test. The results of the independent sample *t*-test showed that the mean score of purchasing intentions in the competence-oriented group was significantly lower than that in the warmth-oriented group (M_competence_ = 4.93, M_warmth_ = 5.44, t = −3.573, *p* = 0.000). It can be seen that different green brand anthropomorphism showed obvious differences in influencing purchasing intentions; that is, compared with competence-oriented, warmth-oriented can significantly positively influence purchasing intentions, which once again verified H2.

#### 4.3.3. Test of Mediating Effect

Study 2 used the PROCESS plug-in in SPSS25.0 to test the mediating effect of psychological distance again. Model 4 was selected for bootstrap analysis, and repeated sampling was set to 5000 times under the 95% confidence level. The specific test results are shown in Table 9. The total effect of green brand anthropomorphism on purchasing intentions was statistically significant (*p* = 0.000), and the confidence interval of the total effect (0.231, 0.797) did not contain 0. The statistical index of the direct effect was significant (*p* = 0.024), and its confidence interval (0.042, 0.597) did not contain 0. The path coefficient of the direct effect was 0.405, which verified the main effect again. The statistical indicator of the indirect effect of psychological distance is significant (*p* = 0.006 < 0.05), and the confidence interval (0.080, 0.349) does not contain 0. Therefore, psychological distance plays a partial mediating role, which verifies H3 again.

#### 4.3.4. Test of Moderating Effect

Study 2 uses two-way ANOVA analysis to test the moderating effect of product attributes in the main effect of this study. In the test, purchasing intentions were selected as the dependent variable, and green brand anthropomorphism and product attributes were selected as the fixed factors, and the test results of the effect between subjects were obtained. The test results show that there is a significant interaction effect between green brand anthropomorphism and product attributes (F (1, 277) = 53.085, *p* = 0.000). Product attributes play a moderating role in the process of green brand anthropomorphism affecting purchasing intentions, and H4 is established.

**Figure 2:**
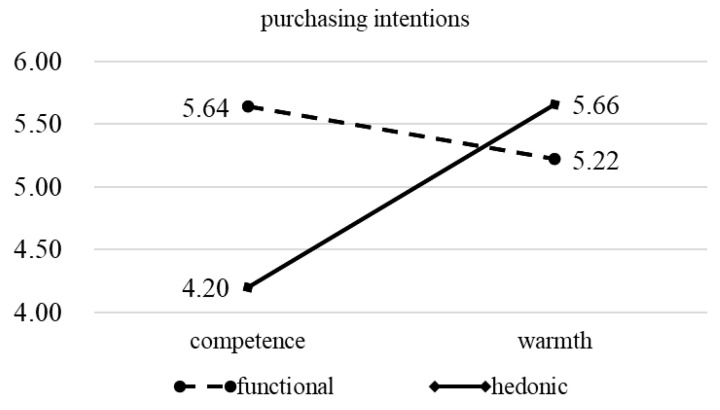
Moderating effect of product attributes (purchasing intentions as DV).

In the next step, independent sample *t*-tests were conducted to examine the differences in the impact of different green brand anthropomorphism on purchasing intentions for functional and hedonic attributes. The moderating effect of product attributes in the research model is shown in Figure 2. The results show that for functional attributes, competence-oriented is more likely to improve consumers’ purchasing intentions (M_competence_ = 5.64, SD = 0.96, M_warmth_ = 5.22, SD = 1.32, t = 2.180, *p* = 0.031). For hedonic attributes, warmth-oriented is more effective in improving purchasing intentions (M_competence_ = 4.20, SD = 0.93, M_warmth_ = 5.66, SD = 1.09, t = −8.558, *p* = 0.000). Therefore, H4a and H4b are verified.

#### 4.3.5. Test of Moderated Mediating Effect

Study 2 uses ANOVA to test whether the interaction effect of green brand anthropomorphism and product attributes on psychological distance is significant. Specifically, Study 2 took green brand anthropomorphism and product attributes as fixed factors and psychological distance as the dependent variable. The results showed that there was a significant interaction effect between green brand anthropomorphism and product attributes (F (1, 277) = 30.578, *p* = 0.000), which initially indicated that product attributes moderated the impact of green brand anthropomorphism on psychological distance.

**Figure 3:**
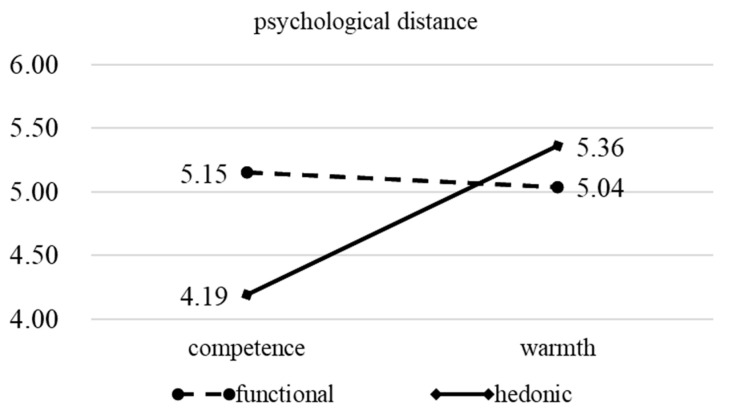
Moderating effect of product attributes (psychological distance as DV).

The independent sample *t*-test was further adopted, and the results showed that for functional, there was no significant difference in the impact of competence-oriented and warmth-oriented on psychological distance (M_competence_ = 5.15, SD = 0.57, M_warmth_ = 5.04, SD = 1.09, t = 0.767, *p* = 0.445, see Figure 3). For hedonic attributes, warmth-oriented can significantly shorten the psychological distance between green brands and consumers more than competence-oriented (M_competence_ = 4.19, SD = 1.10, M_warmth_ = 5.36, SD = 1.04, t = −6.474, *p* = 0.000, see Figure 3).

To further test the mediating effect with moderation, Study 2 used the PROCESS plug-in, selected Model 7 for bootstrap analysis, and set the repeated sampling to 5000 times under the 95% confidence level. Table 10 shows the results of the moderated mediation effect test. The results show that the confidence interval is (0.214, 0.823), which does not contain 0, and the effect value is 0.474, indicating that there is a moderating mediating effect with psychological distance as the mediating variable. Specifically, in the hedonic group, the confidence interval was (0.203, 0.727), which did not include 0, and the effect value was 0.432, indicating that psychological distance played a mediating effect. In the functional group, the confidence interval is (−0.168, 0.061), which contains 0, indicating that the mediating effect of psychological distance is not significant. In summary, product attributes play a moderating effect in the process of green brand anthropomorphism affecting psychological distance, and there is a moderating mediating effect, so H5 is verified. All hypothesis test results are shown in Table 11.

## 5. General Discussion

This study examines the influence mechanism of green brand anthropomorphism on consumers’ green purchasing intentions and finds that anthropomorphism can significantly improve purchase intention compared with non-anthropomorphism, which confirms the positive role of brand anthropomorphism in green marketing. This is consistent with the findings of Han et al. (2023), who pointed out that in sustainable product advertising, anthropomorphism cues can effectively improve consumer preferences. Furthermore, we found that warmth-oriented anthropomorphism had a significantly better effect on promoting green purchase intention than competence-oriented anthropomorphism, which confirmed the “Primacy-of-Warmth Effect” and highlighted the dominant value of the warmth dimension in green brand anthropomorphism. Sehgal et al. (2023) also found that green brand anthropomorphism positively affected repurchase intention through brand warmth and psychological ownership, which confirmed the promotion value of anthropomorphism to green consumption, which was consistent with the main findings of this study. Similarly, the study by El Hedhli et al. (2023) also confirmed that when visual influencers promote products, warmth is more likely to increase consumers’ willingness to recommend and follow than competence, because warmth can trigger emotional resonance, while the emotional experience gain of competence is limited. However, some studies have pointed out that in some consumption situations, consumers show more preference for competence-oriented anthropomorphism (F. Liu et al., 2022). The reason for this difference may be that their research emphasizes low-safety products, whose safety and functional needs have a higher selection priority, rather than the unified characteristics of utility and environment highlighted by green products.

This study also found that psychological distance plays a partial mediating role between green brand anthropomorphism and purchasing intentions; that is, anthropomorphism shortens the psychological distance between consumers and green brands, thereby stimulating purchase intention. The mediating effect of psychological distance is in line with the basic cognitive mechanism of anthropomorphism. Stinnett et al. (2013) proposed that anthropomorphism can narrow the psychological distance between consumers and brands. This study extends this mechanism to the green brand scenario and confirms its mediating value in the influence of anthropomorphism on purchase intention, which is in line with the view of Sehgal et al. (2023) that “anthropomorphism enhances the emotional connection between brands and consumers”.

In addition, the effect of product attributes on green brand anthropomorphism has also been confirmed. Both functional and hedonic attributes can influence consumers’ willingness to engage in sustainable consumption, as confirmed by existing research (C. A. Lin et al., 2023). Functional attributes emphasize practical environmental protection capabilities (Bai et al., 2024), and competence-oriented anthropomorphism can convey professional reliability. On the contrary, hedonic attributes focus on emotional experience (Issock et al., 2023), and warmth-oriented anthropomorphism can strengthen emotional resonance. This matching logic is more in line with the value orientation of the product itself. This result is consistent with the findings of Y. Zhang and Wang (2023), whose research on AI products shows that the anthropomorphic appearance of utilitarian products affects purchase intention through perceived usefulness, while the anthropomorphic appearance of hedonic products affects purchase intention through perceived entertainment. Qin et al. (2025) also had similar findings, revealing that when functional green products are matched with competence anthropomorphism, the professional and efficient characteristics of competence anthropomorphism and personal practical interests form a synergy when the advertisement simultaneously conveys egoistic appeals, which can significantly improve the willingness to purchase.

## 6. Conclusions

### 6.1. Research Findings

This study focuses on the influence mechanism of green brand anthropomorphism on consumers’ purchasing intentions, divides green brand anthropomorphism into two types, competence-oriented and warmth-oriented, and introduces the mediating variable psychological distance and the moderating variable product attributes (functional vs. hedonic) to carry out the research, and the main findings are as follows: (1) Compared with non-anthropomorphism, green brand anthropomorphism can significantly improve purchasing intentions. (2) The promotion effect of warmth-oriented on purchasing intentions is significantly better than that of competence-oriented. (3) Psychological distance plays a mediating role between green brand anthropomorphism and purchasing intentions. (4) Product attributes regulate the effect of green brand anthropomorphism. Functional attributes are suitable for competence-oriented, and hedonic attributes are suitable for warmth-oriented. (5) Product attributes also regulate the effect of green brand anthropomorphism on psychological distance. For functional attributes, there is no significant difference between the effects of competence- and warmth-oriented on psychological distance; for hedonic attributes, warmth-oriented can significantly shorten the psychological distance between green brands and consumers.

### 6.2. Theoretical Contributions

First, we subdivided the types of green brand anthropomorphism and expanded the application boundary of SCM. Existing studies on brand anthropomorphism mostly focus on the effect of a single dimension or discuss the difference between warmth and ability in the general brand context, but there are relatively few studies on the type segmentation and effect comparison of green brand anthropomorphism. Because green brands carry special value attributes such as pro-social and environmental protection, the action logic of their anthropomorphism strategies is essentially different from that of ordinary brands. Existing studies have not fully revealed the differentiated effects of different anthropomorphism types in this specific scenario. Based on SCM, this study divides green brand anthropomorphism into two types, competence-oriented (conveying professional environmental protection technology reliability) and warmth-oriented (conveying environmental protection feelings), and systematically explores the differences between the two in improving consumers’ green purchase intention. This segmentation breaks through the limitations of the existing research on the “generalization” of green brand anthropomorphism and clarifies the dominant advantage of warmth-oriented anthropomorphism in the green consumption scenario. This finding also provides new empirical support for the application of SCM in the field of green consumption. Previous studies have verified the applicability of the “Primacy-of-Warmth Effect” in the virtual influencer scenario (El Hedhli et al., 2023) but have not involved the special field of green brands. This study combines SCM with the core value of green brands, which not only broadens the application scenario of the theory but also provides a new analytical framework for the refined study of green brand anthropomorphism. At the same time, the type segmentation of this study responds to the call of Sharma and Rahman (2022) for “deepening the research on the types of anthropomorphism”, incorporating the type differences in green brand anthropomorphism into the research on green consumption, and enriching the cross-research content of the two fields of green consumption and brand anthropomorphism.

Second, this work introduces the mediating variable of psychological distance, which improves the influence mechanism of green brand anthropomorphism. Existing studies on the mechanism of green brand anthropomorphism have focused on paths such as psychological ownership and green perceived value (Sehgal et al., 2023; Qin et al., 2025) and have paid less attention to the role of psychological distance. Based on CLT and SOR theory, this study innovatively introduces psychological distance into the research model, revealing the transmission mechanism of psychological distance in the influence of green brand anthropomorphism on purchase intention. This finding provides a new psychological perspective for understanding how anthropomorphism affects green consumption decisions. Unlike the psychological ownership emphasized by Sehgal et al. (2023), the mediating effect of psychological distance in this study focuses more on the shortening of the “cognitive–emotional” distance between consumers and brands, which is more in line with the consumer trust barrier that green brands often face due to the opacity of environmental protection information. By integrating CLT and SOR theory, we have achieved an effective cross-theory integration, breaking through the limitations of the single-theory support of existing research, providing a more comprehensive theoretical perspective for the mechanism research of green brand anthropomorphism, and enriching the application scenarios of psychological distance in the field of green consumption.

Third, we further verified the moderating effect of product attributes and clarified the boundary of the moderated mediation. Although previous studies have focused on the impact of product types on anthropomorphism effects, they have focused on AI products or ordinary consumer goods and have not discussed the matching logic between product attributes and anthropomorphism types for the environmental protection characteristics of green brands. The adaptation logic of anthropomorphism strategies for green brands with different core value appeals due to their functional and hedonic attributes needs to be clarified. This study introduces product attributes as a moderating variable, verifies the matching effect of brand anthropomorphism and product attributes, and confirms that product attributes have a significant moderating effect on the path of green brand anthropomorphism affecting psychological distance, forming a complete moderated mediation model. This finding not only expands the application scope of product attributes in the field of green consumption but also clarifies the boundary conditions of the mediating effect of psychological distance, and clarifies the applicable scenario boundary of green brand anthropomorphism strategies. At the same time, the verification of the moderated mediation effect enriches the deep understanding of the mechanism of green brand anthropomorphism, indicating that the mediation effect of psychological distance is not universal but depends on the matching relationship between product attributes and anthropomorphism types. This finding not only improves the theoretical system of the moderating effect of product attributes but also provides more detailed theoretical support for the precise implementation of green brand anthropomorphism strategies.

Last, regarding environmental sustainability, this work’s contribution primarily manifests through indirect pathways at the behavioral level. Specifically, green brand anthropomorphism enhances emotional closeness by reducing psychological distance, thereby increasing consumers’ propensity toward green choices in specific purchasing situations. These micro-level green decisions, when accumulated over time, can influence environmental sustainability outcomes (V. Boivin & Boiral, 2022). Correspondingly, the behavioral science contributions of this study lie not only in proposing and testing psychological distance as a key mechanism, but also in providing a replicable operationalization process for anthropomorphism types. This includes the development of competence/warmth-oriented text and images, manipulation checks, and control of confounding factors, thereby enhancing the testability of anthropomorphism research within the green consumption context at the methodological level. More importantly, green consumption often involves stronger eco-friendly and ethical motivations (D. Yang et al., 2015). This context reveals a clearer mechanism for anthropomorphism in value- and responsibility-driven decision-making, thereby enriching the understanding of brand anthropomorphism theory regarding situational boundaries and operational pathways.

### 6.3. Practical Implications

Our research also provides some practical implications. First, green brand anthropomorphism types can be accurately matched with product attributes to implement differentiated green marketing strategies. This simple sustainable communication requires minimal cognitive effort from consumers, making it crucial for promoting sustainable behavior (Acuti et al., 2022). For functional green products (such as energy-saving home appliances, environmentally friendly building materials, and water-saving equipment), we need to focus on competence-oriented anthropomorphism strategies to create a professional and reliable image, such as environmental protection technology experts and efficient energy-saving guardians. Through technical analysis videos, environmental protection data visualization, professional certification endorsements, etc., we can highlight the environmental protection technology advantages, functional reliability, and practical value of the products, such as “saving 500 degrees of electricity in 3 years”, and “professional environmental protection certification”. In addition, in product packaging, instructions, and advertisements, anthropomorphic images play the role of technical consultants, detailing the working principle of the product, the calculation method of environmental protection results, and the solution of the use scenario, which is in line with the core appeal of consumers for functional products, which is “rational and practical”. For hedonic green products (such as eco-friendly beauty products, eco-tourism services, and organic food gift boxes), a warmth-oriented anthropomorphism strategy should be adopted. Warm and friendly anthropomorphic images (such as natural partners and green life stewards) should be designed to convey the brand’s environmental protection story (such as “sourced from natural raw materials, protecting the skin and the earth”), while encouraging users to share their emotional experiences and contextualize the content of emotional resonance, highlighting the brand’s caring and altruistic qualities. In addition, in the communication, we should focus on emotional connection, such as letting anthropomorphic images tell the environmental protection original intention behind the product development and the vision of going to green life with consumers, which meets the core needs of emotional experience contained in hedonic attributes. If the product attributes are ambiguous, such as green household products that have both functional and hedonic attributes, the warmth-oriented anthropomorphism strategy should be adopted first. This choice is in line with the finding in this study that “warmth-oriented as a whole can better enhance the willingness to purchase green products”.

Second, enterprises can optimize the presentation and communication of green brand anthropomorphism to strengthen brand awareness and emotional connection. In the design of anthropomorphic images, it is necessary to combine the brand’s environmental protection positioning and the preferences of the target customer group, and AI design tools and professional teams can be used to create differentiated visual symbols. For example, the anthropomorphism images of functional green products can use cool colors with simple and neat styling elements to highlight the sense of professionalism. The images of hedonic products can use warm colors, matched with round and friendly modeling elements, to convey a sense of warmth. At the same time, the anthropomorphism design adopts the first-person tone (Han et al., 2023). For example, competence-oriented can use “I use professional technology to protect every degree of energy saving”, and warmth-oriented can use “I stand shoulder to shoulder with you to integrate green into life”, using simple and straightforward language to strengthen the perception of brand anthropomorphism.

Third, companies can innovate interactive forms to shorten the psychological distance between brands and consumers. With the help of technology, immersive experiences can be created to strengthen psychological connections. For example, AR technology can be used to develop interactive functions. Consumers can scan product packaging or advertising posters with their mobile phones to watch the environmental protection production process, raw material traceability, and product environmental protection effect simulation guided by anthropomorphic images, and intuitively perceive the brand’s environmental protection strength and shorten the cognitive distance. For smart green products, such as smart and environmentally friendly home appliances, anthropomorphic interactive assistants can be embedded in the product APP to provide personalized environmental protection usage suggestions, environmental protection achievement check-ins, and other functions, so that anthropomorphic images can become companions of consumers’ green life. It is also possible to build a community interaction scene to deepen emotional bonding. By establishing an official brand community, anthropomorphic images can be used as community hosts to regularly launch environmental protection topic discussions (such as sharing your green life tips), environmental protection task challenges (such as 7-day low-carbon check-ins), and other activities to encourage consumers to share green life stories related to products, strengthen consumers’ sense of participation and belonging, and further shorten the emotional distance between brands and consumers (Sehgal et al., 2023).

### 6.4. Limitations and Future Research

This work also has some limitations. First, this study used a professional questionnaire platform to produce and distribute questionnaires and simulated experimental scenarios online. Although it has tried its best to combine graphic and text simulation scenarios and replaced different experimental stimulus materials and subjects to carry out experiments, there are still some differences from the real shopping environment, and the display content is only in the form of static graphics and text. In the future, online quasi-experiments and offline field experiments can be fully combined, and the form of green brand anthropomorphism display can be enriched. Secondly, the experimental stimuli in this study mainly involve the categories of energy-saving household appliances, environmentally friendly daily necessities, and sustainable fashion products. In the future, more categories of experimental stimuli can be selected to increase the universality of the research conclusions. Finally, this study mainly measures consumers’ willingness to buy, but there is still a gap between willingness to buy and behavior in real life. In future research, we can further test the influence mechanism of green brand anthropomorphism on purchase behavior and try to divide anthropomorphism into different types from other perspectives. We can also add variables such as negative word-of-mouth of green brands to explore whether it will cause negative effects.

## Figures and Tables

**Table 1:** The correspondence between research hypotheses and studies.

Research Hypothesis	Purpose of Testing	Study
H1	Compared to non-anthropomorphism, green brand anthropomorphism effectively enhances purchasing intentions	Study 1
H2	Different anthropomorphism types (competence vs. warmth) lead to distinct green purchasing intentions	
H3	Mediating role of psychological distance	
H4	Moderating role of product attributes on green brand anthropomorphism on purchasing intentions	Study 2
H5	Moderating role of product attributes on green brand anthropomorphism on psychological distance (moderation-mediated model)	

**Table 2:** Demographic information of Study 1.

Type	Item	Value	Quota Proportion (%)
Gender	Male	105	47.09
	Female	118	52.91
Age	0–20	5	2.24
	21–30	112	50.22
	31–40	82	36.77
	41–50	18	8.07
	51–60	6	2.69
Educational level	High school and below	7	3.14
	Associate degree	13	5.83
	Bachelor’s degree	99	44.39
	Master’s degree	104	46.64

**Table 3:** Convergent validity analysis of Study 1.

Construct	Item	Standardized Loading	CR	AVE
Psychological distance	PD1	0.895	0.921	0.744
	PD2	0.880		
	PD3	0.864		
	PD4	0.810		
Purchasing intentions	PI1	0.830	0.880	0.649
	PI2	0.830		
	PI3	0.707		
	PI4	0.843		

**Table 4:** Discriminant validity analysis of Study 1.

	Psychological Distance	Purchasing Intentions
Psychological distance	0.863	
Purchasing intentions	0.606	0.806

**Table 5:** Test of the mediating effect of psychological distance (Study 1).

Mediating Effect	Coeff	SE	t	*p*	LLCI	ULCI
Total effect (c)	0.894	0.214	4.180	0.000	0.471	1.316
Direct effect (c’)	0.416	0.152	2.741	0.007	0.116	0.716
Indirect effect (a × b)	0.478	-	-	0.007	0.167	0.821

**Table 6:** Demographic information of Study 2.

Type	Item	Value	Quota Proportion (%)
Gender	Male	121	43.06
	Female	160	56.94
Age	0–20	12	4.27
	21–30	143	50.89
	31–40	91	32.38
	41–50	22	7.83
	51–60	13	4.63
Educational level	High school and below	8	2.85
	Associate degree	16	5.69
	Bachelor’s degree	152	54.09
	Master’s degree	105	37.37

**Table 7:** Convergent validity analysis of Study 2.

Construct	Item	Standardized Loading	CR	AVE
Psychological distance	PD1	0.882	0.862	0.612
	PD2	0.764		
	PD3	0.727		
	PD4	0.747		
Purchasing intentions	PI1	0.892	0.931	0.773
	PI2	0.868		
	PI3	0.843		
	PI4	0.908		

**Table 8:** Discriminant validity analysis of Study 2.

	Psychological Distance	Purchasing Intentions
Psychological distance	0.782	
Purchasing intentions	0.394	0.879

**Table 9:** Test of the mediating effect of psychological distance (Study 2).

Mediating Effect	Coeff	SE	t	*p*	LLCI	ULCI
Total effect (c)	0.514	0.144	3.573	0.000	0.231	0.797
Direct effect (c’)	0.320	0.141	2.266	0.024	0.042	0.597
Indirect effect (a × b)	0.194	-	-	0.006	0.080	0.349

**Table 10:** Test result of moderated mediating effect.

Effect Types		Effect	SE	t	*p*	Boot LLCI	Boot ULCI
Direct effect		0.320	0.141	2.226	0.024	0.042	0.597
Mediating	functional	−0.041	0.057	-	-	−0.168	0.061
	hedonic	0.432	0.135	-	-	0.203	0.727
moderated mediating effect		0.474	0.158	-	-	0.214	0.823

**Table 11:** Summary of Hypothesis Test Results.

Content of the Test	Hypothesis	Supported or Not
Compared to non-anthropomorphism, green brand anthropomorphism effectively enhances purchasing intentions	H1	Supported
Different anthropomorphism types lead to distinct green purchasing intentions	H2	Supported
Mediating role of psychological distance	H3	Supported
Moderating role of product attributes on green brand anthropomorphism on purchasing intentions	H4	Supported
Moderating role of product attributes on green brand anthropomorphism on psychological distance	H5	Supported

## Data Availability

The raw data supporting the conclusions of this article will be made available by the authors on request.

## Supplementary Materials

The following supporting information can be downloaded at: https://www.mdpi.com/article/10.3390/bs16030316/s1, File S1: Experimental Material for Study 1; Experimental Material for Study 2.
