# Supplementary material for: Show Me Competence or Make Me Feel Warm: The Impact of Green Brand Anthropomorphism on Consumers’ Purchasing Intentions
Source: Behav Sci (Basel). 2026 Feb 25;16(3):316. doi: 10.3390/bs16030316 (PMC13023449; doi:10.3390/bs16030316)
Supplement: Supplementary file 1 [file behavsci-16-00316-s001.zip › behavsci-4135474-supplementary.pdf]

Supplementary Materials

Experimental Material for Study 1

The refrigerator as stimuli

Pilot Test 1: Word Validation

**Competence Phrases**	**Warmth Phrases**
high efficiency	walk alongside you
energy-saving	care for your family's health
quiet operation	keep food's freshness and flavor
adaptive operation saves you electricity	sleep soundly
intelligently adjusting the temperature	stand guard in the kitchen corner
capacity-optimized compartment	accompany home's tranquility and comfort
reduce carbon emissions	love always within reach
innovative foaming technology	thoughtful drinking companion
low-carbon polyurethane materials	warmth of sunshine
better preservation	love for life

Pilot Test 2: Image Validation

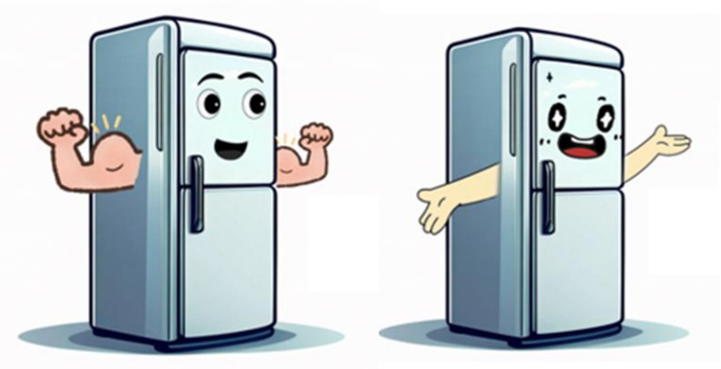

Pilot Test 3: Same-copy and Different-images Validation

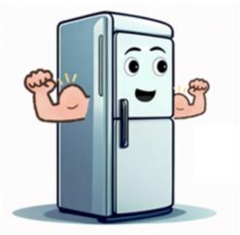

EcoFreeze is committed to providing more environmentally friendly home appliance solutions. We continuously reduce our environmental impact through material selection, production processes, and packaging design, while empowering consumers to make greener choices through transparent information disclosure. Choose EcoFreeze to effortlessly embrace sustainable consumption in daily life.

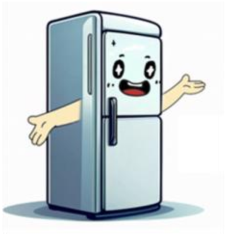

EcoFreeze is committed to providing more environmentally friendly home appliance solutions. We continuously reduce our environmental impact through material selection, production processes, and packaging design, while empowering consumers to make greener choices through transparent information disclosure. Choose EcoFreeze to effortlessly embrace sustainable consumption in daily life.

Pilot Test 4: Different-copy and Different-images Validation

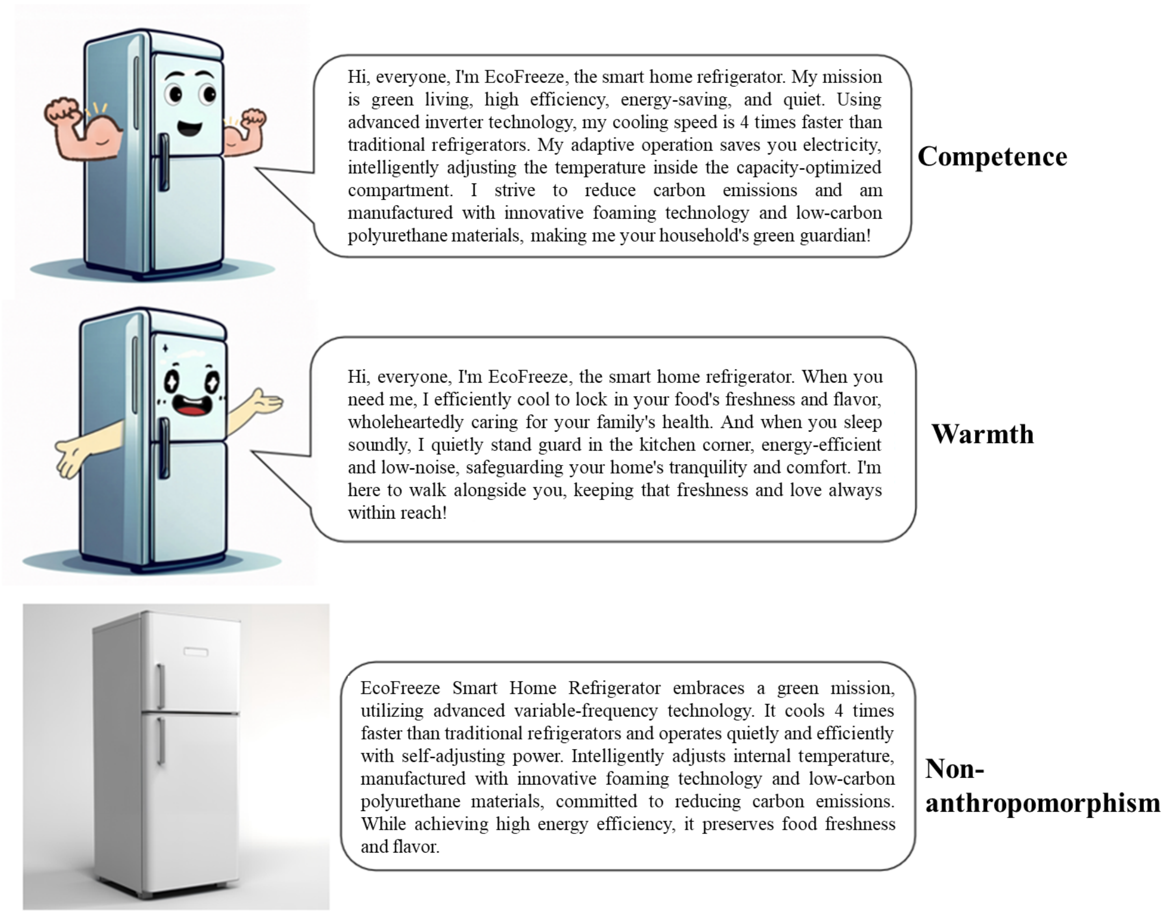

Experimental Material for Study 2

The water cup as stimuli

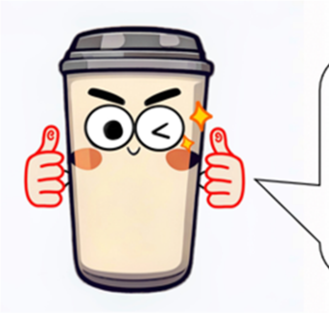

Hi, everyone, I'm "Little Wheat," the wheat straw water cup. Made from natural wheat straw, I'm durable and shatter-resistant. While ensuring I'm renewable, biodegradable, and pollution-free, I also help keep you safe from health risks. I feature a spill-resistant rim with a leak-proof seal, and my smooth interior is easy to clean, making your drinking experience more convenient. I look forward to providing you with an excellent drinking experience!

Competence

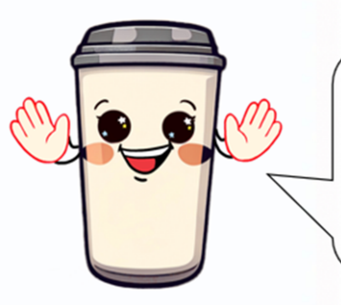

Hi, everyone, I'm "Little Wheat," the wheat straw water cup. I firmly believe nature's gifts are the ultimate choice. I bring the freshness of fields and the warmth of sunshine to be your thoughtful drinking companion, cherishing your health and embracing your love for life. Choose me, and with every sip, you'll feel the purity and warmth from nature!

Warmth

The perfume as stimuli

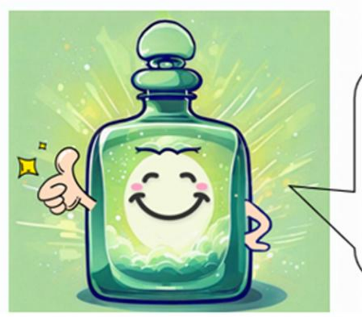

Hi, everyone, I'm NatureScent, the eco-friendly perfume. My ingredients are biodegradable, pure natural, safe, and chemical-free, gently caring for your skin. My scent has the power to relax your nerves, relieve stress, and regulate your mood. It awakens your senses, making you feel incredibly comfortable. My packaging rejects plastic and is fully recyclable and reusable!

Competence

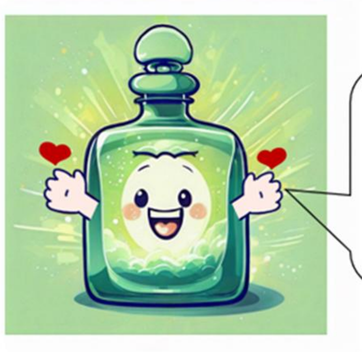

Hi, everyone, I'm NatureScent, the eco-friendly perfume. "No" to chemical additives, and every drop is nature's profound gift, pure and precious. A gentle spray instantly transports you to a lush forest, where floral scents abound and warmth envelops you, telling tales of spring, helping you find resonance with nature amidst busy city life, and feel the beauty of life!

Warmth
